# Improving Robot Localization Using Doppler-Based Variable Sensor Covariance Calculation

**DOI:** 10.3390/s20082287

**Published:** 2020-04-17

**Authors:** Bibiana Fariña, Jonay Toledo, Jose Ignacio Estevez, Leopoldo Acosta

**Affiliations:** Computer Science Departament, University of La Laguna, Avd Fco Sanchez, Fisica y Matematicas, 38200 La Laguna, Spain; bfarinaj@ull.es (B.F.); iestevez@ull.es (J.I.E.); lacosta@ull.es (L.A.)

**Keywords:** odometric, sensor fusion, localization, robotics

## Abstract

This paper describes a localization module for an autonomous wheelchair. This module includes a combination of various sensors such as odometers, laser scanners, IMU and Doppler speed sensors. Every sensor used in the module features variable covariance estimation in order to yield a final accurate localization. The main problem of a localization module composed of different sensors is the accuracy estimation of each sensor. Average static values are normally used, but these can lead to failure in some situations. In this paper, all the sensors have a variable covariance estimation that depends on the data quality. A Doppler speed sensor is used to estimate the covariance of the encoder odometric localization. Lidar is also used as a scan matching localization algorithm, comparing the difference between two consecutive scans to obtain the change in position. Matching quality gives the accuracy of the scan matcher localization. This structure yields a better position than a traditional odometric static covariance method. This is tested in a real prototype and compared to a standard fusion technique.

## 1. Introduction

In a modern robot, the localization sub-module is based on a combination of multiple sensors. Each sensor has its strengths and weaknesses, and only when properly combined can they yield robust localization in every situation.

One of the most used fusion techniques is the Kalman filter [1,2] and its variants. A Kalman filter is a statistical method that fuses information from sensors based on their accuracy. Very accurate sensors with small covariances can be fused with less accurate ones. All the sensors contribute to the localization; even relatively imprecise sensors can be used to improve the final estimated position of the wheelchair. The key step in the Kalman filter is to characterize the sensors’ covariance, so the fusion can be done properly to obtain an accurate final localization at every point.

**Figure 1:**
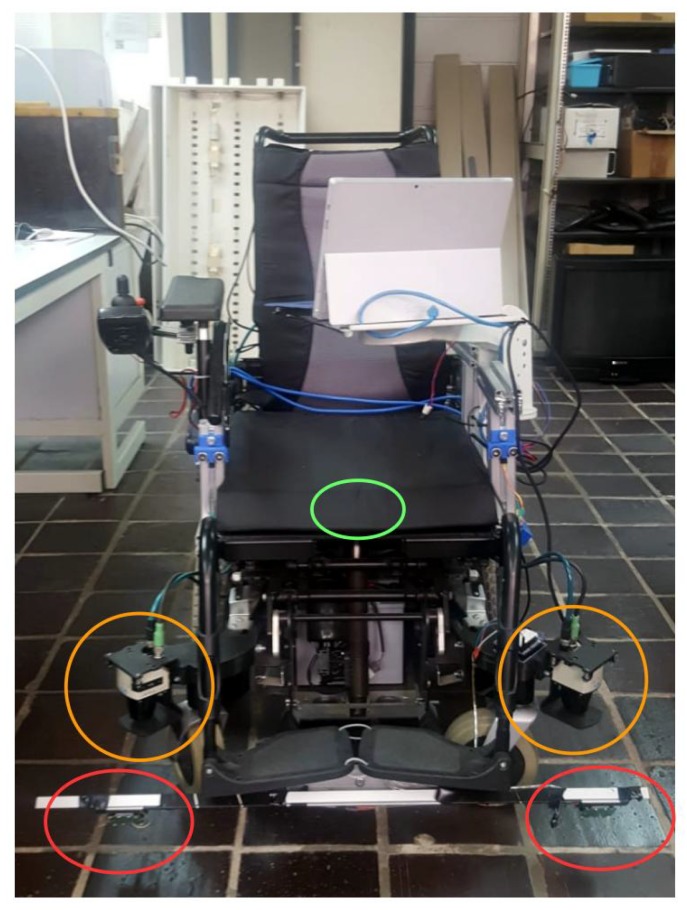
The intelligent wheelchair prototype with the set of lidar, odometer, IMU and Doppler sensors.

This paper presents a localization system for an intelligent wheelchair, the prototype is shown in Figure 1. The wheelchair is designed to transport people with severe disability, helping users move with less effort and improving their quality of life. This intelligent wheelchair is controlled by the user, indicating the final desired pose. A set of sensors will detect obstacles and reconstruct the environment [3] in order to navigate safely and autonomously [4,5]. The wheelchair will make the low-level decisions, avoiding obstacles and selecting the best path to arrive at the desired location [3]. In order to achieve this task, the localization subsystem has a key role in the movement process. This paper presents the localization subsystem for the wheelchair, which is based on a sensor fusion scheme.

The wheelchair localization subsystem consists of the following sensors:

- *Odometry*. Wheel odometry uses incremental encoders coupled to the motors. The system captures the movement of the wheels in real time. It is one of the main sensors for a robotic prototype and the basis for any localization system [6]. The position and orientation is obtained by integrating wheel displacement. Odometry is an accurate sensor for short distances, but the error becomes unlimited over long distances.
- *IMU*. Inertial Measurement Unit (IMU) uses a set of sensors to estimate inertial magnitudes. In this case an electronic gyroscope, which measures angular speed, is integrated to estimate orientation. The IMU also has an electronic compass to determine the north direction, but the behavior of this sensor can be erratic indoors due to magnetic interference, and it is not used in the localization module.
- *Lidar*. Light Detection and Ranging (LIDAR), with the scan matching technique [7], calculates the difference between two consecutive laser scans to estimate the movement between them. This difference is converted to an incremental displacement of the wheelchair, similar to conventional odometry.

The set of sensors used in the wheelchair is shown in Figure 1. Circled in orange are the lidars, a pair of Sick TiM 551, valid for indoor and outdoor use, with a maximum range of 8 m, an angular resolution of 1 degree, a field of view of 270 degrees, and a measurement frequency of 15 Hz. In red are the HB100 Doppler effect sensors, described in Section 4. In green is the IMU unit, located under the seat. It is an MPU9250 which includes an accelerometer, gyroscope and magnetometer in a single chip.

This paper describes the fusion process for the sensors presented above. Each sensor’s accuracy is characterized by a variable covariance, so the fusion algorithm can weigh sensor importance depending on the environment. The method for obtaining the variable covariance of each sensor, the fusion algorithm and the tests for comparing variable and static covariance localization, done with a real prototype, are presented in the sections below.

This paper is organized as follows. Section 2 contains a study of previous research using Doppler based localization, which is compared to the topic of this paper. In Section 3, the standard fusion algorithm for a static covariance sensor is described. In Section 4, the method for estimating variable covariance based on an odometric sensor and Doppler speed radar is introduced. Section 5 describes the experiments with the algorithm presented and provides a comparison to a standard algorithm in a real environment with a working prototype. Section 6 presents the conclusions of our work.

## 2. Previous Work

Modern robot localization is based on a multiple-sensor fusion algorithm. One of the most widely used algorithms is the Kalman filter, where each sensor in the filter is characterized by a covariance and the filter is applied in order to obtain a robust localization [8,9]. The covariance depends on the sensor’s physical characteristics and is usually calculated based on data or tuned by hand [10]. Other algorithms, such as Monte Carlo localization, use a set of particles to sample the probability distribution of the robot’s position [11,12,13]. In this case, the particles evolve based on sensor data and covariance, so covariance is a key parameter in any localization algorithm.

Covariance is one of the main ways to characterize sensor accuracy, so variable covariance is necessary in order to obtain a robust algorithm, as in [14], where multiple sensing is integrated using a Kalman filter and variable covariance based on errors for each sensor, or in [15], where a fusion scheme for multiple sensors in a driver assistance system is presented, with each sensor being characterized by a variable covariance.

Intelligent wheelchairs are a specific case of an autonomous robot, and an extensive bibliography is available on the topic. The main difference between a standard mobile robot is safety. The robot is designed to transport disabled people, meaning each movement must be comfortable and safe. Ref. [16] provides a survey of different classical intelligent wheelchair projects, including a description of technologies and interfaces. In [17], a 2017 review of wheelchair technology is collected. The main obstacle detection and localization sensors used nowadays in wheelchairs are odometers, lidars, IMU and cameras, the information from which is fused to yield a final position. The main area of research in intelligent wheelchairs is the human machine interface so as to simplify user control. In [18], the sensors in an intelligent wheelchair are used to compensate for uncertainty in the commands given to the wheelchair by an eye tracking device. In [19], a specific wheelchair prototype with a sensory system based on odometry, ultrasound, and a camera is presented.

Previous works used different techniques to fuse the information coming from the sensors and obtain robust and accurate real-time localization. In the intelligent wheelchair case, an accurate localization is the first step in navigating safely, so the system must be robust. In this paper, an innovative method based on sensor fusion and variable covariance calculation is used to improve localization accuracy. Odometry is a key part of the localization subsystem of any robot, though the accuracy of this kind of sensor is usually very hard to measure. Odometry is affected by systematic and non-systematic errors, so systematic ones can be included in a static covariance calculation; however, non-systematic errors cannot be included in a static covariance and they can produce significant errors in the final position. The problem with non-systematic errors is that they may appear unexpectedly (for example, when the robot passes over an unexpected object on the ground, or the robot’s wheels slip), and they can cause large position errors, so a robot that relies on odometry is not reliable. In this paper, other low-cost sensors, such as Doppler radar speed sensors, are used to measure odometry information accuracy and change their covariance to improve the final localization accuracy.

The application of radars to localization is not widespread, only a few works are presented in the bibliography. In [20], a continuous wave rotating radar and a scan matching algorithm between two consecutive scans are used to estimate the position change between two consecutive scans. In [21], a scan matching technique based on Iterative Closest Point ICP over radar data and a Kalman filter are used in order to integrate the data for vehicle localization. The most similar work is [22], where the Doppler data of all the angles is used to obtain the ego vehicle’s speed, and the directional information of each angle in the radar scan is used to detect landmarks and estimate the change in the vehicle’s position based on scan matching algorithm. In all this works, the radar is used as a Lidar, and a scan matching algorithm is used to detect robot movement.

In this work, the data produced by a low cost Doppler radar is used to measure the accuracy of the wheelchair’s odometric sensor. Doppler radar can measure the displacement speed based on the Doppler frequency shift, however the sensor is very noisy, so it is difficult to integrate as independent odometric sensor. However, it can be used as a accuracy sensor, to measure the variable localization quality of a standard odometric system in form of a variable covariance matrix. As far as authors concern, this fusion strategy is not previously used. The experiments presented in Section 3 and Section 5 show that this fusion can reduce significantly the accumulative localization errors when some of the sensors fails.

## 3. Localization with Odometric Static Covariance

In this section, we present the basic localization algorithm based on fusing static covariance sensors. This algorithm is usually the first step in the localization system of any robot, and for simple mobile robots it may be enough. However, for an intelligent wheelchair, the algorithm has to be robust, meaning some improvements must be made.

Covariance is a key parameter in the information from a sensor and must be specified for each sensor in a localization system. In the localization system of the wheelchair, lidars are used as a localization source based on a scan matching algorithm [7] which measures the difference between two scans to obtain the change in the robot’s position. Lidar scan matching covariance can be calculated based on the similarity between two consecutive scans [23]. If the current and previous scans are very similar, the accuracy of the position change calculation will be high. The change in the robot’s position is obtained based on the movement of similar representative points between two scans. The change between the points in the first and the second scans gives the final incremental movement of the robot. If all the points are correlated by the expected movement, the covariance will be small, indicating an accurate incremental movement. If the environment is not well defined or has fuzzy points, if other obstacles are moving in the lidar field of view or not enough references are available within the range of the lidar, the number of representative points will be small and the result will not be accurate. The incremental movement will thus have a large covariance, resulting in a bad localization. It is also possible that the difference between two consecutive scans will not match, so the movement between two matches cannot be estimated. This will generate an error and a very high covariance, indicating a bad incremental localization. The accuracy of the laser scan matching algorithm is thus characterized by a variable covariance. This allows the fusion algorithm to weigh its contribution to the final position every measurement.

(1) $$\begin{array}{c} L_{ok}^{l}=\frac{2*\pi *r_{l}*count_{k}^{l}}{encRes};\;L_{ok}^{r}=\frac{2*\pi *r_{r}*count_{k}^{r}}{encRes}\\ ▵\theta_{k}=\frac{\left(L_{ok}^{l}-L_{ok}^{r}\right)}{Wheeldisp}\\ ▵d_{k}=\frac{\left(L_{ok}^{l}+L_{ok}^{r}\right)}{2}\\ \theta_{k+1}=\theta_{k}+▵\theta_{k}\\ X_{k+1}=X_{k}+▵d_{k}cos\left(\theta_{k+1}\right)\\ Y_{k+1}=Y_{k}+▵d_{k}sin\left(\theta_{k+1}\right) \end{array}$$

The equations that govern an encoder odometric-based sensor are shown in Equation (1). These equations are valid when $L_{ok}^{l}$ and $L_{ok}^{r}$ are small, so the increment angle ▵θ and the displacement $▵d_{k}$ are also small. In Equation (1), $L_{ok}^{l}$ and $L_{ok}^{r}$ are the displacement of left and right wheels in a certain time period based on the odometric sensor, $r_{l}$ and $r_{r}$ are the left and right wheel radii, $count_{k}^{l}$ and $count_{k}^{r}$ are the number of encoder ticks that the electronics receive in time period *T*, encRes is the encoder resolution in one turn. $▵\theta_{k}$ is the variation in the wheelchair angle in the time period, and depends on Wheeldisp, which is the distance between the robot’s wheels. ▵d is the change in distance traveled based on wheel displacement, and $X_{k}$ and $Y_{k}$ are the robot’s position in Cartesian coordinates.

Wheel odometry and IMU covariance are frequently more difficult to adjust. Sensor information in a good or bad localization scenario does not change, and a static average covariance is usually assigned to these sensors [9]. This covariance underestimates odometry localization when the wheels travel in a perfect environment, but this does not usually introduce localization errors. The main disadvantage appears when a non-systematic error occurs. This can happen when a wheel slips in any area of the robot’s path. The odometry system cannot detect this error and its covariance will remain constant. The sensor fusion algorithm would consider odometric movement as valid data with a small covariance. This can lead to a bad localization of the robot’s final position. The IMU sensor measures the rotational speed, but as with any other analog sensor, white noise is included in the measurement, so the orientation angle based on the integration of this noisy sensor has a very low accuracy. A high covariance is assigned to the IMU sensor due to its low accuracy, so the sensor will be only used when other sensors are failing. In a standard localization implementation based on laser scan matching, encoder odometry and an IMU, the laser scan matching will have a variable covariance, while wheel odometry and IMU have a static covariance. The traditional way to optimize the fusion filter is to tune the weight of the odometry covariance to obtain the best position, but non-systematic errors can lead to robot delocalization.

(2) $$\begin{array}{c} x_{k+1}=f\left(x_{k},u_{k},w_{k}\right)\\ z_{k}=h\left(x_{k},v_{k}\right) \end{array}$$

**Figure 2:**
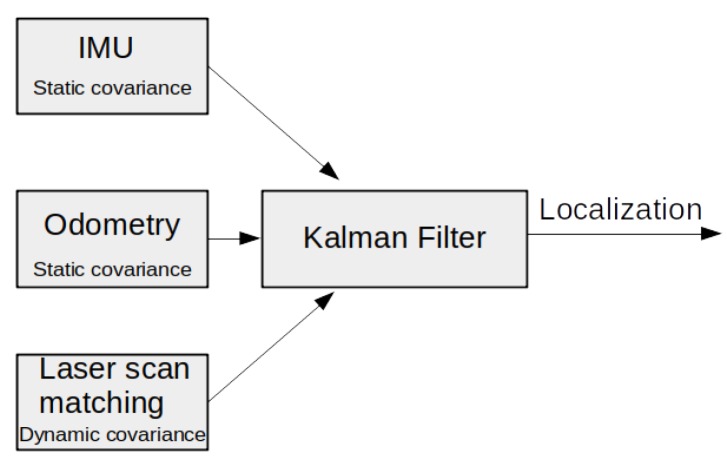
Schematic localization with odometric static covariance.

Figure 2 shows the basic localization diagram based on a Kalman filter [1,2]. The localization module receives sensors and covariances as inputs, and gives a localization estimation as the output. The Kalman filter combines sensor information and a system model in order to estimate the current state. Equation (2) presents a model of the localization system [24], where $x_{k+1}$ is the space state we want to estimate, $z_{k}$ are the actual measurements of the system, in our case, sensor information, f() is the model state space matrix, which depends on the previous state $x_{k}$, the model input $u_{k}$ and the process noise $w_{k}$, and h() is the observation model which converts internal states to measurement outputs and depends on measurement noise $v_{k}$.

The robot movement model is based on a 2D omni-directional model Equation (3). The model relies on the assumption that velocity does not change over a period of time, meaning it is a constant speed model. The model’s inputs are unknown; the only information available is the wheel encoders that are used for the odometry and are input as a measurement to the Kalman filter. A theoretical model can be constructed based on the motor transfer function, but it will provide the same information as odometry, because the same sensors are used to calculate motor speed. So a standard model without inputs is used based on a kinematic model derived from Newtonian mechanics. The model is shown in Equation (4), where *T* is the time period, and ${\dot{X}}_{k},{\dot{Y}}_{k},{\dot{\theta }}_{k}$ are the speeds of every variable.

(3) $$\begin{array}{c} x_{k+1}=Ax_{k}+w_{k}; \end{array}\;\left[\begin{array}{c} X_{k+1}\\ Y_{k+1}\\ \theta_{k+1}\\ {\dot{X}}_{k+1}\\ {\dot{Y}}_{k+1}\\ {\dot{\theta }}_{k+1} \end{array}\right]=\left[\begin{array}{cccccc} 1 & 0 & 0 & T & 0 & 0\\ 0 & 1 & 0 & 0 & T & 0\\ 0 & 0 & 1 & 0 & 0 & T\\ 0 & 0 & 0 & 1 & 0 & 0\\ 0 & 0 & 0 & 0 & 1 & 0\\ 0 & 0 & 0 & 0 & 0 & 1 \end{array}\right]\left[\begin{array}{c} X_{k}\\ Y_{k}\\ \theta_{k}\\ {\dot{X}}_{k}\\ {\dot{Y}}_{k}\\ {\dot{\theta }}_{k} \end{array}\right]+w_{k}$$

The state of the system is shown in Equation (3), which relies on the Cartesian position $\left(X_{k},Y_{k}\right)$, orientation $\theta_{k}$ and speed. A Kalman filter will be used to estimate the current state $x_{k}$. This gives good results for smooth, non-linear functions, such as those described by the robot, if the sample period is small.

(4) $$\begin{array}{c} {\tilde{x}}_{k+1}=Ax_{k}\\ P_{k+1}=A_{k}P_{k}A_{k}^{T}+R_{k}\\ K_{k+1}=P_{k}H_{k}^{T}{\left[H_{k}P_{k}H_{k}^{T}+Q_{k}\right]}^{-1}\\ {\tilde{x}}_{k+1}\left(+\right)={\tilde{x}}_{k}+K_{k}\left[z_{k}-H_{k}{\tilde{x}}_{k}\right]\\ P_{k+1}\left(+\right)=\left[I-K_{k}H_{k}\right]P_{k} \end{array}$$

Equation (4) shows the Kalman filter algorithm, where $R_{k}$ is the covariance of the model noise $w_{k}$, which is set to a high value to give more importance to the measurement than to the model evolution. $Q_{k}$ is the covariance of each measurement (lidar, encoder odometry and IMU) $u_{k}$. This covariance can be variable, as in the case of the lidar, for example, or static, for odometric and IMU. $P_{k}$ is the covariance for the estimated space state $x_{k+1}$ starting in the initial position covariance $P_{0}$. $H_{k}$ is the mapping matrix between the sensor output and the model. This matrix represents the measurement in the space state vector $x_{k}$ for each sensor. The Kalman filter provides an estimation based on the state space $x_{k}$ and an accuracy estimation $P_{k}$ for every sensor measurement received. The final accuracy $P_{k}$ is better than the individual sensors.

**Figure 3:**
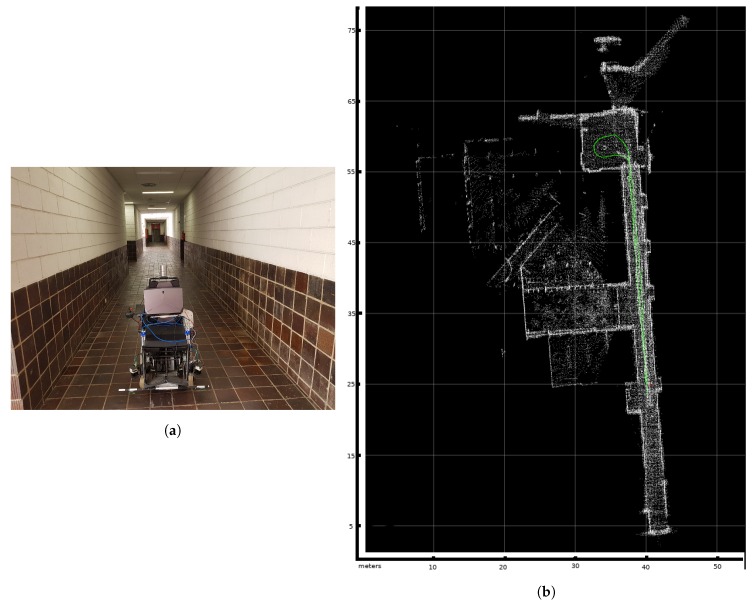
The tests are conducted in a long corridor with a turnaround area at the end. (**a**) The wheelchair in the corridor where the tests are carried out with the Velodyne lidar installed. (**b**) The test area and the path traveled by the wheelchair (green line). Each square of the grid represents 10 m.

The tests are conducted in a long corridor with a turnaround area at the end, Figure 3. Along the route, different speeds and different kinds of floors are used to ensure the experiments cover many circumstances. The ground truth is obtained using a Velodyne HDL 32 lidar. This sensor consists of 32 lidars with different angles to scan the transverse area. Each laser has an accuracy of 2 cm. The lidar information is used to obtain the route traveled and the map of the environment using a LOAM slam algorithm [25]. This algorithm has exhibited very high localization accuracy when used with a Velodyne lidar, enough to be considered as ground truth. Figure 3a shows a picture of the wheelchair with the Velodyne installed. The Velodyne is only used to obtain the ground truth. Figure 3b shows the map generated based on the LOAM algorithm, with the route taken during the experiment shown with a green line.

**Figure 4:**
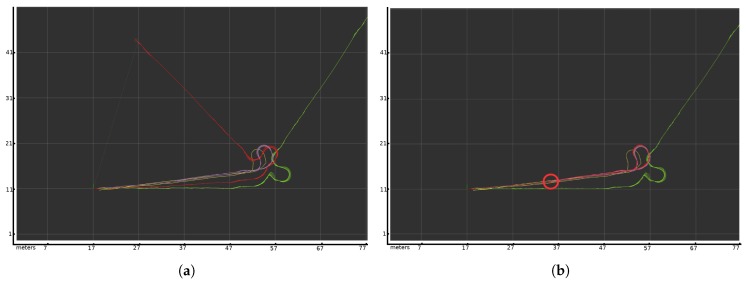
Change in wheelchair’s position in the corridor. The grid represents a resolution of 10 m. The green line corresponds to the position reported by the odometry sensor. The purplr line is the position reported by the laser scan matcher, and the red one shows the result of fusing the odometry, IMU with static covariance and laser scan matcher with variable covariance. The yellow line represents the ground truth displacement according to the Velodyne sensor. (**a**) Test where static odometric covariance takes precedence over variable laser scan matching covariance in a corridor with a turn. (**b**) Variable laser scan matching covariance takes precedence over static odometric covariance in the same scenario. The red circle marks the final position.

Figure 4 shows an example of wheelchair localization in the specific environment using static covariance for IMU and odometry and variable covariance for laser scan matching. The setting is a long corridor with a turn at the end. The corridor is a very complicated test for the laser scan matcher because the laser can only see two walls and it has very few reference points, which makes it very difficult to calculate the robot’s displacement. Besides, the floor where the robot turns is slippery, so the wheels move but the wheelchair does not. Odometry indicates that the robot is turning, but the wheels are slipping. The covariance is static, so the sensor fusion algorithm considers odometry information as valid. This situation generates a non-systematic error which affects the final localization.

Figure 4 shows the evolution of this experiment with IMU and odometric static covariance. The route begins with good localization reported by all the sensors, but when the wheelchair arrives at the turnaround area, the wheels slip and generate an orientation error of more than 90 degrees, as shown in the green plot. This slippage also introduce erroneous displacement to the odometric output localization. However, in the slippage area, laser localization is quite accurate, as shown in the purple plot. Upon returning to the initial point, the laser scan matching algorithm cannot obtain an accurate localization in the corridor due to the absence of representative points. The position obtained from laser scan matching indicates less displacement than actual, thus generating a position error.

Figure 4 shows the behavior depending on the odometry covariance weight. In the case of an odometry overweight scenario, Figure 4a, the distance traveled is correct, but the orientation error includes the wheel slip and the odometry error getting a final error of 34 m. In the other case, Figure 4b shows an underweight odometry scenario: the turn is made correctly based on the laser, but in the corridor, the distance traveled is less than actual. The final point is marked in the plot with a red circle. This localization reports a displacement of about 18 m away from the actual end position.

This result shows that, in this experiment, it is not possible to properly adjust the weights to solve the localization problem with static covariance values. The weight can prioritize odometry or laser scan matching, but the final position is usually a combination of the error introduced by each sensor, as shown in Figure 4. The only solution is a variable covariance, where the accuracy of odometric data is weighted and its covariance changes according to its results.

The IMU data is also used in the combination, but its data is less accurate than that of the other sensors, so its effect is residual compared to laser scan matching or odometry. This sensor just manages the orientation results when the other two sensors fail. As with any other sensor, it will improve the final results but the system usually works well with a manually tuned static covariance.

## 4. Variable Odometric Covariance

A way to improve the final localization accuracy is to increase the quality of odometric data. The objective is to use other sensors to detect non-systematic odometry errors in order to eliminate or reduce their effect.

### 4.1. Doppler Sensor

**Figure 5:**
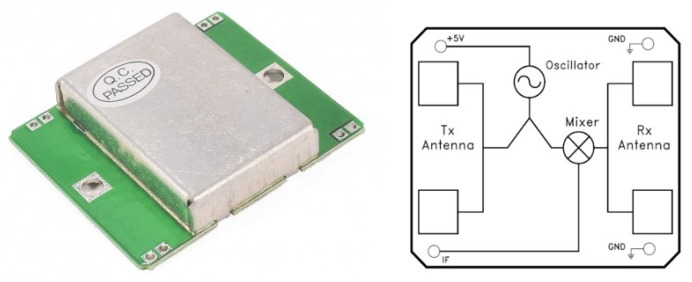
The low-cost RF Doppler sensor used as the odometry covariance sensor.

In this paper, low-cost Doppler effect sensors are used to measure wheelchair speed as an alternative to odometry. If the expected distance traveled by the wheel, as measured by the Doppler effect, is not equivalent to the odometric distance, the covariance of the odometric measurement will increase, indicating a possible odometric error. The Doppler effect is measured using the HB-100 low-cost RF Doppler sensor shown in Figure 5. The module consists on a Dielectric Resonator Oscillator (DRO), a microwave mixer and a patch antenna (see Figure 5 right). The module is a low-power radio device (LPRD) emitting a frequency of 10.525 Ghz.

(5) $$f_{d}=\left(\frac{c\pm v_{r}}{c\pm v_{s}}\right)f_{0}$$

The Doppler effect (or the Doppler shift) is the change in frequency or wavelength of a wave in relation to an observer who is moving relative to the wave source. Equation (5) describes the Doppler effect, where $f_{d}$ is the Doppler output frequency, $v_{r}$ is the velocity of the receiver in relation of the medium, $v_{s}$ is the velocity of the source relative to the medium, *c* is the speed of light and $f_{0}$ is the frequency of the transmitted wave. In the case of a moving Doppler sensor placed on a robot, $v_{r}=v_{s}$, so the frequency change of the system is shown in Equation (5), where $v_{r}$ is the speed of the robot, which can move forward or in reverse.

**Figure 6:**
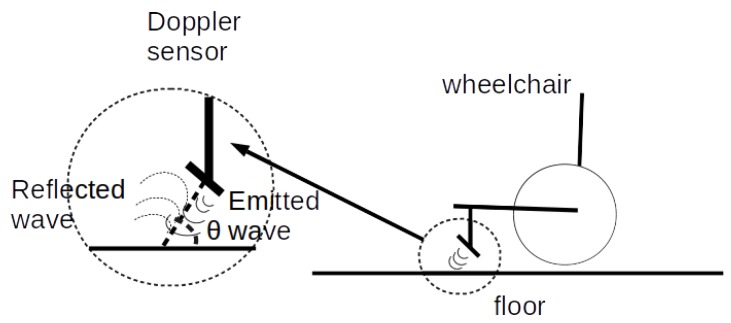
Working principle of the Doppler sensor installed in the wheelchair.

The HB100 module emits a high frequency RF signal, and receives the reflected signal. The frequencies of the emitted and received signals are mixed in a superheterodyne style circuit, yielding an Intermediate Frequency (IF), as the difference between the emitted and received frequencies. Equation (6) shows this difference, where $f_{d}$ is the received frequency and $f_{0}$ the emitted frequency. If the sensor is not moving, the emitted and reflected signals will have the same frequency, so the output of the IF mixer will be 0. If the sensor is moving, the reflected signal will be affected by the Doppler effect. The difference between them will be a small signal of a few hertz. This Doppler shift output comes from the IF terminal when movement is detected. The magnitude of the Doppler shift is proportional to transmitted energy reflected and depends on the angle of emission. This magnitude is in the microvolt (μV) range. A high-gain, low-frequency amplifier is connected to the IF terminal in order to amplify the Doppler shift to a measurable level. This low-frequency output is measured using custom designed electronics for measuring between 0.1 and 1000 Hertz. The frequency of the Doppler shift is proportional to velocity of motion. When walking, a typical human generates a Doppler shift below 100 Hz. Figure 5 shows the sensor installed in the wheelchair, with one Doppler effect sensor in front of each wheel in order to measure each wheel’s speed. Figure 6 shows the working principle of the sensor installed in the wheelchair. The Doppler sensor is placed about 10 cm above the floor oriented at 45 degrees. The emitted signal is reflected by the ground and part of that signal is received again by the receiver antenna. The sensor’s output is not affected by the environment or people, it only depends on the reflection from the floor.

Equation (6) shows the device frequency output, where $F_{d}$ is the output Doppler frequency after the mixer and the subtraction, $v_{r}$ is the speed of the robot, $f_{0}$ is the transmission frequency of 10.525 Ghz, *c* is the speed of light and θ is the angle between the direction of motion and the axis of the module.

(6) $$F_{d}=f_{d}-f_{o}=2v_{r}\frac{f_{0}}{\left(c-v_{r}cos\left(theta\right)\right)}cos\left(\theta \right)\approx 2v_{r}\frac{f_{0}}{c}cos\left(\theta \right)$$

**Figure 7:**
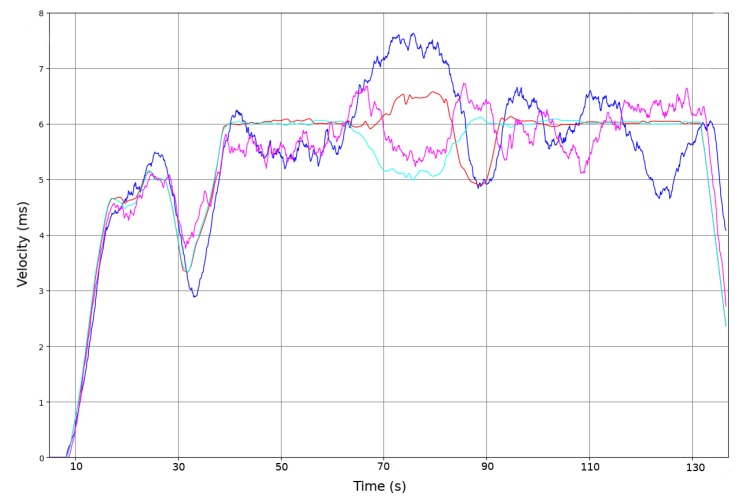
Velocity calculation based on Doppler and odometric sensor in a non-slip area. The X-axis is time in seconds, and the Y-axis is velocity m/s. The red and light blue lines are wheel speed based on the odometric sensor. The pink and blue lines represent the speed based on the Doppler sensor.

In order to test the Doppler effect sensor and the custom electronic circuit, a set of experiments was conducted to calibrate the quality of the Doppler speed sensor. Two HB-100 sensors were placed on either side of the wheelchair to measure the displacement based on Doppler shift. Figure 1 shows the two HB-100 sensors in front of the left and right wheels. The frequency received is transformed to speed and distance traveled based on Equation (6). The tests were run in the part of the corridor where the wheels do not slip, Figure 4, and compared to the odometric output. Figure 7 shows the speed based on the Doppler and odometry sensors. The red line represents the left odometric wheel speed, the blue line the left Doppler wheel speed, the light blue line the right odometric wheel speed and the pink line the right Doppler wheel speed. As Figure 7 shows, the results of both sensors are quite similar, and represent the same movement for each wheel of the wheelchair. The Doppler speed measurement is noisy and depends on the quality of the reflected signal, but it represents the movement speed accurately.

**Figure 8:**
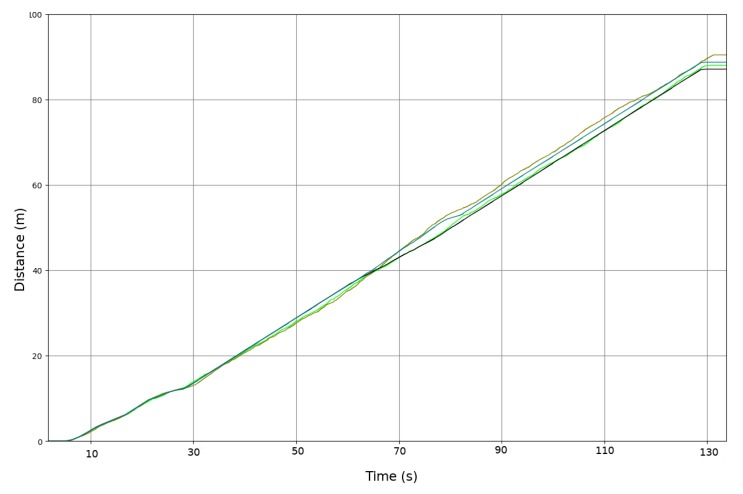
Calculation of total distance traveled based on Doppler and odometric sensors in a non- slip area. Black and blue displacement based on odometric sensor. Green and brown displacement based on radar speed sensor.

Figure 8 shows the total distance traveled by each wheel. The black and blue lines are the displacement of the left and right wheels based on the odometric sensor. The green and brown lines are the same distance traveled, but based on the Doppler effect sensor described in this paper. As Figure 8 shows, the total error after 90 m of travel is less than 2 m. The Doppler sensor speed measurement does not exhibit enough quality for a Doppler-only odometric system. It is noisier than an encoder-based odometric system; however, it is a good sensor for detecting non-systematic errors in the encoder-based odometric system, as next section shows.

### 4.2. Odometric Variable Covariance

**Figure 9:**
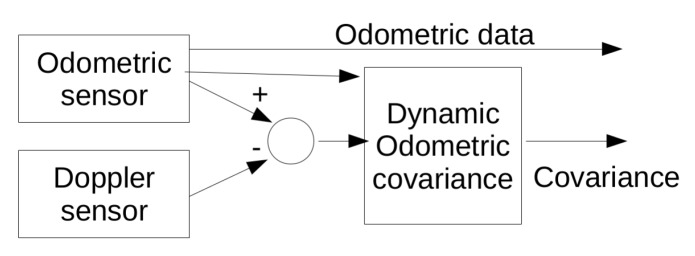
Diagram of the variable covariance change based on Doppler and odometric data comparison.

The wheelchair speed is obtained using two different sensors with different physical supports: encoders attached to the wheel motors and Doppler shift radar. The inconsistency between the results of these two sensors can be used to estimate sensor accuracy. Figure 9 shows the variable covariance estimation module where the difference between the wheel speeds based on Doppler radar sensor and odometer is used to estimate a variable covariance that will be used in a subsequent filter to fuse with other sensors.

The module obtains the difference between the speeds based on the encoder odometric system and the Doppler effect sensor, integrating the reported speed over time period *T*, Equation (7), where ${▵}_{n}$ represents the difference in length traveled by the *n* (left or right) wheel based on each sensor in the time period, $v_{D}^{n}$ is the speed measured by the Doppler sensor and $v_{O}^{n}$ is the speed measured by the encoder odometry. This difference represents an error between sensors measuring the same parameter. If the difference is large, one or both sensors are not measuring correctly. In this case, the problem is assumed to come from the odometry side. This means that its covariance is increased.

(7) $${▵}_{n}=\int^{T}v_{D}^{n}dt-\int^{T}v_{O}^{n}dt$$

The wheel displacements, $L_{t}^{l}$ and $L_{t}^{r}$, are measured based on two different sensors. In order to reduce noise, an integration window of 500 ms is used to convert Doppler speed into displacement. Encoder speed is less affected by the noise, but the same integration window is applied. This displacement can be characterized by its mean and variance, as shown in Equation (8), where $L_{d_{t}}^{n}$ is the displacement based on the Doppler sensor of the *n* wheel (left or right), $L_{d_{t}}^{n}$ is the wheel displacement based on the odometer sensor for the left or right wheel, α is the reliability between the odometer and Doppler sensors, 0≥α≥1, in our experiments α=0.5 but it can be adjusted based on sensor accuracy, $E(L_{t}^{n})$ is the average distance traveled and $var(L_{t}^{n})$ is the variance of the distance traveled measurement. α parameter is used to tune the accuracy relation between Doppler and wheel odometer sensor. If the wheel odometer has a small resolution, or odometric parameters can’t be estimated accurately, Doppler sensor can be more reliable, and α should be bigger than 0.5, in other cases where odometric sensor has a very high accuracy, α will be less than 0.5. In our experiments, the accuracy relation between odometry and Doppler sensor is compensated, so a value of 0.5 is selected.

(8) $$\begin{array}{c} E\left(L_{t}^{n}\right)=\alpha L_{d_{t}}^{n}+\left(1-\alpha \right)L_{o_{t}}^{n}\\ var\left(L_{t}^{n}\right)=\alpha {\left(E\left(L_{t}^{n}\right)-L_{d_{t}}^{n}\right)}^{2}+\left(1-\alpha \right){\left(E\left(L_{t}^{n}\right)-L_{o_{t}}^{n}\right)}^{2} \end{array}$$

Combining Equation (1) and Equation (8), the estimated variable covariance can be introduced into the odometer model, as shown in Equation (9), where $var(▵\theta_{t})$ is the estimated variable covariance for the change in the orientation angle, and $var(▵d_{t})$ is the variable covariance for the motion of the center of the wheelchair. As expected, the covariance is higher for the angle changes in the wheelchair case, so Wheeldisp is usually less than 1 m. When the wheelchair travels a long distance, this covariance is integrated and the position error grows indefinitely, so an external sensor is needed in order to reset the position and the accumulated error.

(9) $$\begin{array}{c} var\left(▵\theta \right)=\frac{1}{Wheeldisp^{2}}\left(var\left(L_{t}^{l}\right)+var\left(L_{t}^{r}\right)\right)\\ var\left(▵d_{t}\right)=\frac{1}{4}\left(var\left(L_{t}^{l}\right)+var\left(L_{t}^{r}\right)\right) \end{array}$$

**Figure 10:**
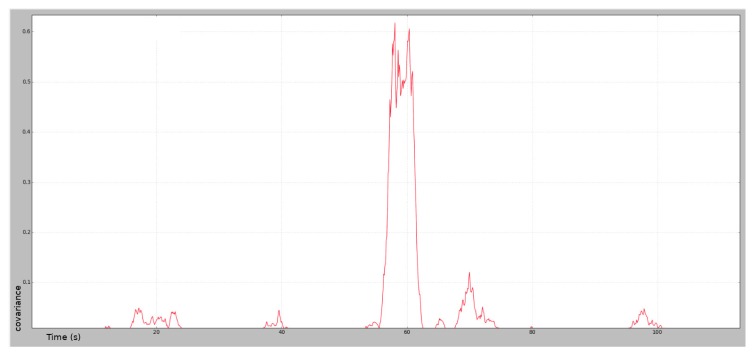
Evolution of the covariance as a comparison between odometric data and Doppler data over time. The X-axis is time in seconds, the Y-axis is covariance.

The odometric covariance calculated according to Equation (8) for the same experiment shown in Figure 4 is given in Figure 10, where the red plot represents the odometer covariance over time. This covariance is usually small, indicating a good localization, meaning the Doppler and odometric sensors are reporting similar values. In the area with slippage, according to Figure 10, the motion measured by the Doppler effect is not correlated by the odometric sensor, so the covariance increases. In this part of the path, the wheels are slipping, so the encoder-based odometer reports a wheel speed that does not correspond to wheelchair movement. The Doppler radar sensor reports a low wheel speed, so it is not affected by the slip. The Kalman filter uses this information to weigh the information from the sensors according to the covariance, and reduces the odometer weight in the slippage area. The variable odometric covariance is used in the next section to obtain a more accurate fusion of the different sensors.

## 5. Results

**Figure 11:**
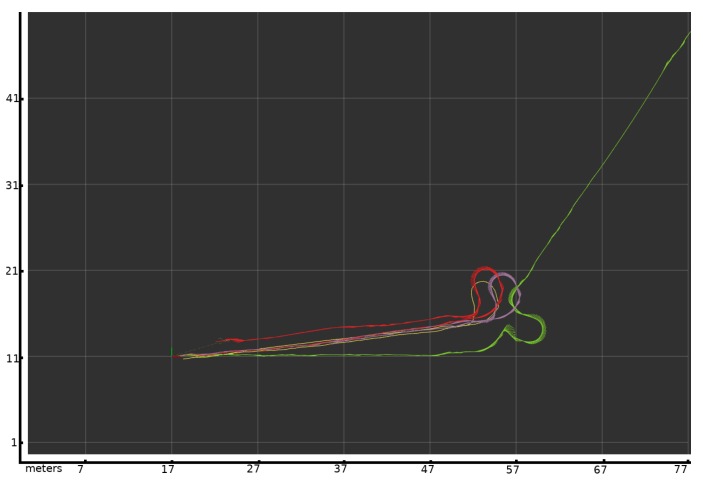
The same experiment as in Figure 4, but using the variable covariance presented in this paper. In the grid, each square represents 10 square meters. The green line is the position based only on odometry. The purple line is the position based on the laser scan matcher algorithm. The red line is the fusion using the variable covariance presented in this paper. The yellow line is the ground truth obtained using the Veldyne sensor.

The results of applying this covariance are presented in Figure 11, which shows the same experiment as in Figure 4, but including the variable covariance change in the sensor fusion. The red plot shows the estimated change in the pose over time using the odometric variable covariance method presented in this paper. At the start, the path is similar for both sensors, so the filter averages both sensors. When the wheelchair arrives at the slippage area, the variable covariance module detects a difference between the odometric and Doppler sensors, so the odometric covariance grows, as shown in Figure 10. This means that the this sensor’s contribution to the final position is reduced due to the error detected by the variable covariance module. The fused localization method follows the scan matching algorithm based on lidar information to obtain an accurate position during the turn. Then, when the wheelchair returns to the corridor, the scan matching algorithm fails, so there are not enough representative points to calculate the displacement in the corridor. The covariance of the odometry sensor is small because the Doppler and encoder sensors give similar information. The fusion algorithm uses the information from the odometric source, meaning it is more reliable. There is a offset of 6 m between the start and end positions due to the cumulative error along the route, but it is an acceptable error after traveling about 150 m. The final error is very small in comparison to the first experiment using static assigned covariance for the fusion algorithm. The experiment shows that variable covariance can compensate for non-systematic and systematic errors and outperforms the static covariance algorithm.

**Figure 12:**
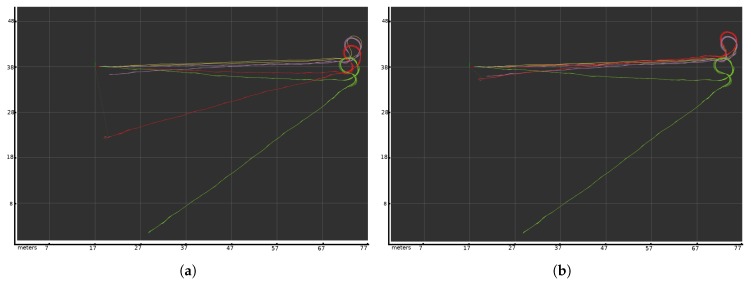
Experiments with static and variable covariance. Green line, path based on the odometry. Purple line, path based on the laser scan matching algorithm. Yellow line, ground truth based on the Velodyne sensor. Red line, the fusion algorithm with static covariance (**a**) and with variable covariance (**b**). Each square on the grid represents 10 m. (**a**) Localization with odometric static covariance set to a medium value. (**b**) Localization with Doppler effect variable covariance in the odometric data.

Figure 12 shows other tests. Figure 12a shows the standard localization method with static covariances when odometric covariance is set to a medium value. This is the standard technique used for robot localization. The wheel slip generates an offset in the odometric localization (green line), introducing part of this error into the system (red line). Figure 12b shows the same scenario, but using the variable covariance adjustment algorithm. In this case, the odometry fault is offset by the small covariance of the odometric system due to the variable covariance technique, meaning the localization algorithm can absorb this error and generate a correct final position.

## 6. Conclusions

In this paper we present a new method to determine the variable covariance of an encoder based on an odometric sensor. This increases the accuracy of the localization system of an intelligent wheelchair.

The variable covariance is based on comparing the Doppler radar speed sensor and encoder odometric information. If the results of both sensors are similar, the covariance will be small, meaning that the data is consistent. If they differ, that means that one of the sensors is reporting an error, so the covariance will be increased to report this to the fusion algorithm. These errors are usually generated by a non-systematic error in the encoder odometric systems, like wheel slip.

The sensor fusion algorithm takes advantage of the variable covariance of each sensor and is able to generate a more accurate output. The results obtained by the localization method based on variable covariance on a real prototype are compared to a standard localization method based on a static covariance. The variable covariance method outperforms the accuracy of the localization of the standard one, yielding a more robust localization system. This system can filter non-systematic errors and provide a robust final position after a long path using only on-board sensors.
